# Supplementary material for: Engineering a riboswitch-based genetic platform for the self-directed evolution of acid-tolerant phenotypes
Source: Nat Commun. 2017 Sep 4;8:411. doi: 10.1038/s41467-017-00511-w (PMC5583362; doi:10.1038/s41467-017-00511-w)
Supplement: Supplementary file 2 — Supplementary Information [file 41467_2017_511_MOESM2_ESM.pdf]

Description of Supplementary Files

File name: Supplementary Information
Description: Supplementary figures and supplementary tables.

File name: Peer review file

SUPPLEMENTARY INFORMATION

Supplementary Figures

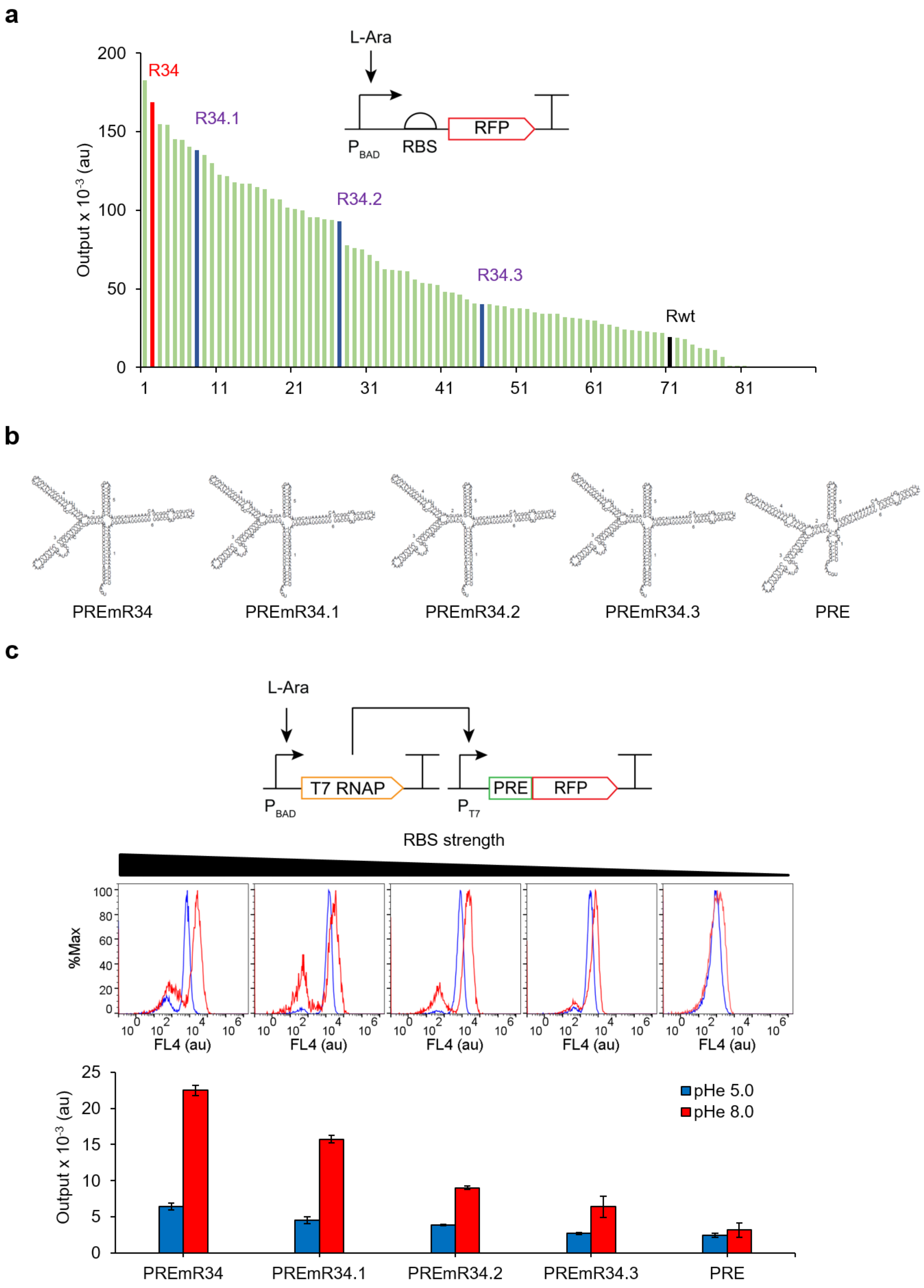

Supplementary Figure 1. Engineering dynamic range of PRE through RBS modifications. (**a**) Translation efficiencies of wild-type RBS of PRE (black bar) with RBS34 variants (red, blue and green bars) generated in this study. (**b**) Predicted OFF structures of PRE, PREmR34, and PREmR34.x variants. (**c**) Output expression at minimal (pHe 5.0) and maximal (pHe 8.0) induction of pH-sensing devices built from PRE, PREmR34, and PREmR34 variants. Data represents mean of three biological replicates.

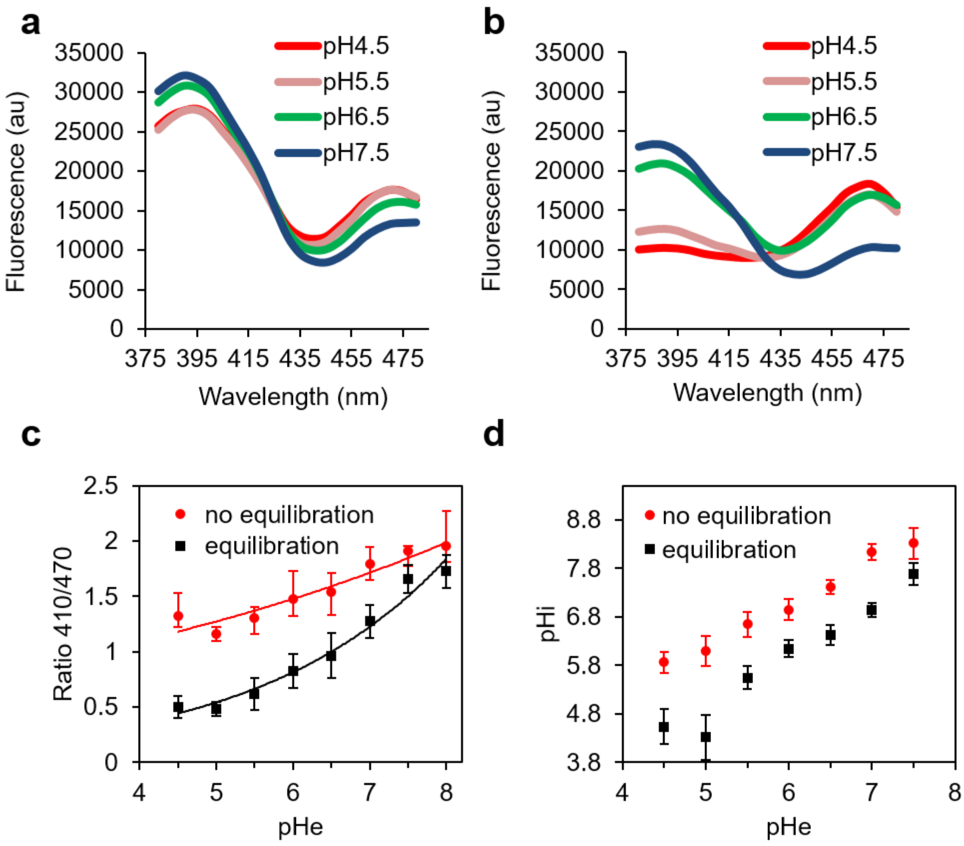

Supplementary Figure 2. Measurement of *E. coli* T10 intracellular pH (pHi). (**a**) Excitation spectrum of *pHluorin2* in TOP10 cells at varying pHe without the use of equilibration reagents. (**b**) Excitation spectrum of *pHluorin2* in TOP10 cells at varying pHe with the use of equilibration reagents (40mM potassium benzoate and 40mM methylamine hydrochloride). (**c**) *In situ* calibration curve of wavelength ratios 410/470 and pH levels. (**d**) Calculated pHi of TOP10 cells at varying pHe. Data represents mean of three biological replicates.

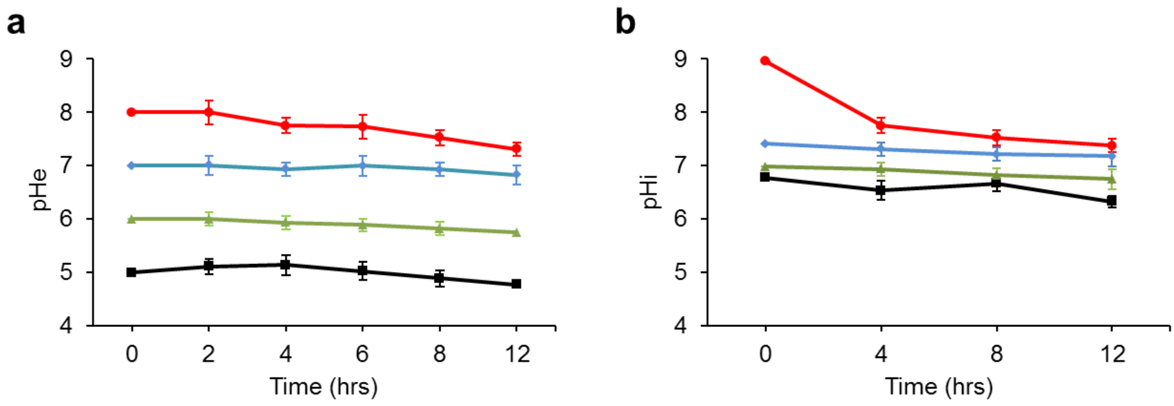

Supplementary Figure 3. Time-course measurement of *E. coli* T10 intracellular pH in different extracellular pH. Mid-log phase T10 seed cultures at pH 7.0 were inoculated into acidified M9 glycerol media and monitored for changes in pHe over time. (**a**) Time-course measurement of pHe in M9 glycerol medium. (**b**) Time-course measurement of T10 pHi in M9 glycerol medium. Each line represents different starting pHe: 5.0 (black), 6.0 (green), 7.0 (blue), 8.0 (red). Data represents mean of three biological replicates.

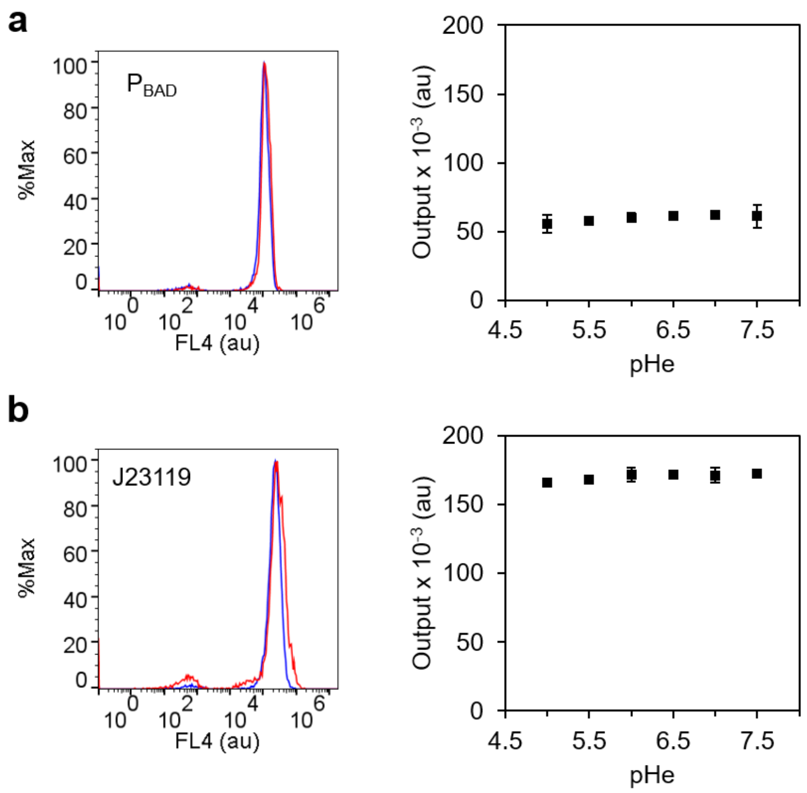

Supplementary Figure 4. Effect of pHe on promoter activities of pBAD and J23119. (**a**) Left: representative flow cytometry at pHe 5.0 (blue) and pHe 8.0 (red) of P_{BAD} promoter. Right: output from P_{BAD} at varied pHe levels (LA 0.01%). (**b**) Left: representative flow cytometry at pHe 5.0 (blue) and pHe 8.0 (red) of J23119 promoter. Right: output from J23119 at varying pHe levels. Data represents mean of three biological replicates.

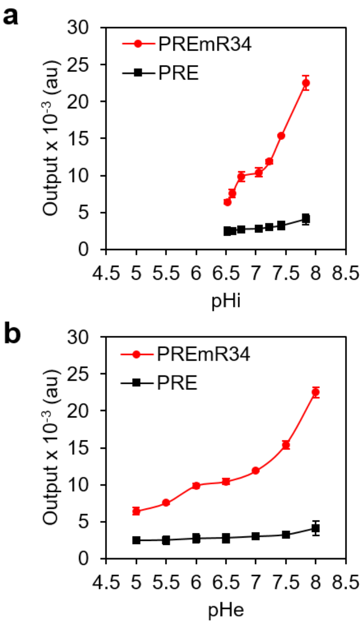

Supplementary Figure 5. Output response of pH-riboswitch circuits built from PRE and PREmR34 plotted against pHi or pHe. (**a**) Output expression plotted against pHi. (**b**) Output expression plotted against pHe.

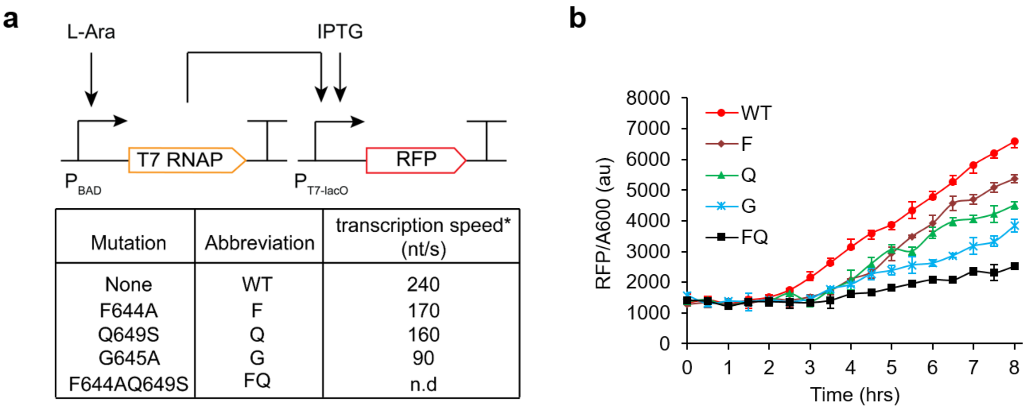

Mutation	Abbreviation	transcription speed* (nt/s)
None	WT	240
F644A	F	170
Q649S	Q	160
G645A	G	90
F644AQ649S	FQ	n.d

Supplementary Figure 6. Transcription efficiencies of different T7RNAP mutants. (**a**) Schematic of genetic constructs used to compare transcriptional activities of T7RNAP mutants. Transcription speed of single amino acid mutants were previously measured *in vitro* by Bonner et al, 1994. (**b**) Dynamic profile of RFP outputs from T7RNAP promoter paired with different T7 RNAP mutants.

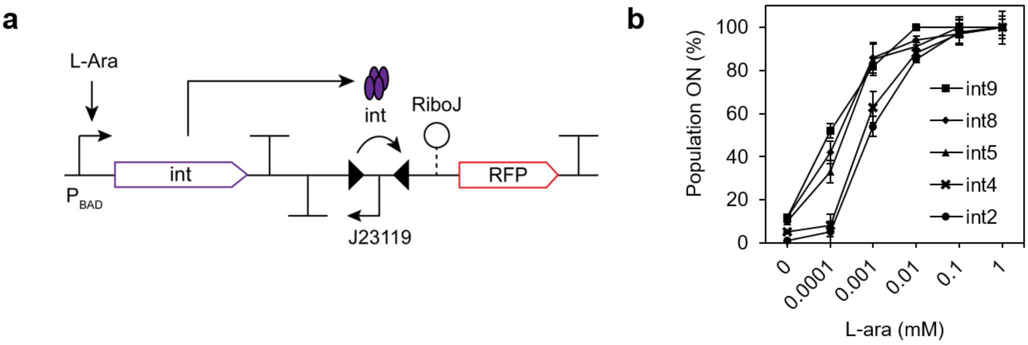

Supplementary Figure 7. Comparison of the dynamic behavior of integrase library. (**a**) Schematic of genetic constructs used to compare the dynamic behavior of integrase library. (**b**) Dynamic behavior of integrase library. The 1-plasmid system was induced with varied L-arabinose levels, and the population of "ON" state cells were measured. The data represents means of three independent experiments.

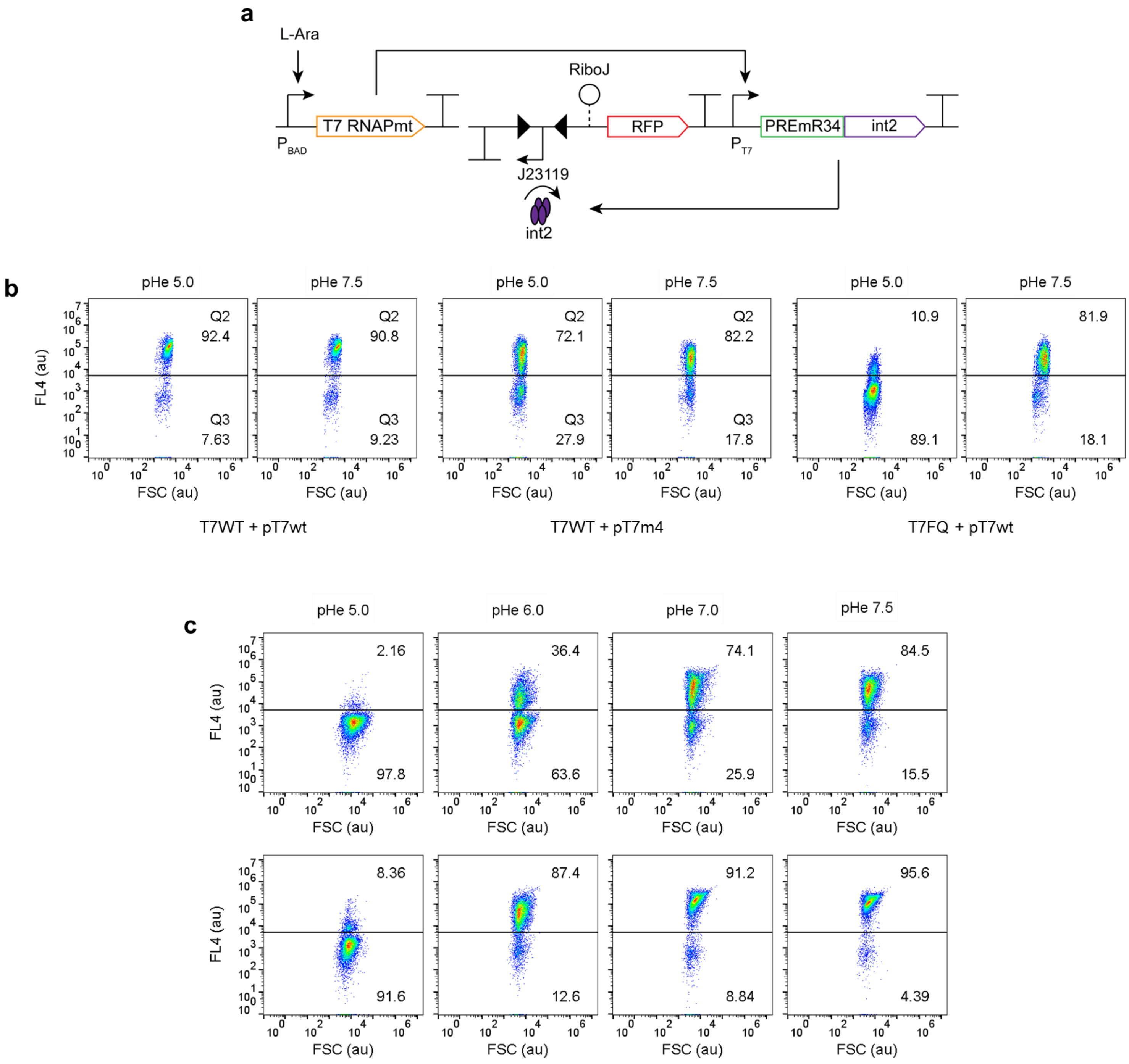

Supplementary Figure 8. Design and characterization of digitalized pH-sensing device. (**a**) Simplified schematic of pH-sensing device. (**b**) Resolving failure mode of digital pH-sensing system by promoter engineering or T7 RNAP engineering. Labels indicate the combination of T7 RNAP and T7 promoter variants. (**c**) Top: Representative flow cytometry of high threshold (low LA, 0.002%) digital pH-sensing system. Bottom: Representative flow cytometry of low threshold (high LA, 0.1%) digital pH-sensing system. Numbers indicate percentage of cell population in bisection.

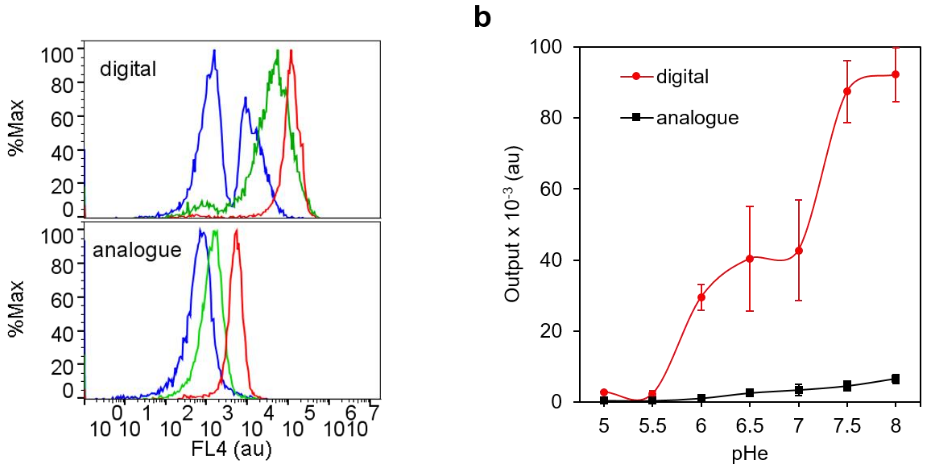

Supplementary Figure 9. Comparing digital and analogue pH-sensing devices. (**a**) Flow cytometry of pH-sensing devices built from FQ mutant at pHe 5.0 (blue), pHe 6.0 (green), and pHe 7.5 (red). (**b**) Geometric mean fluorescence of various pH-sensing devices at varied pHe levels. Data represents mean of three biological replicates.

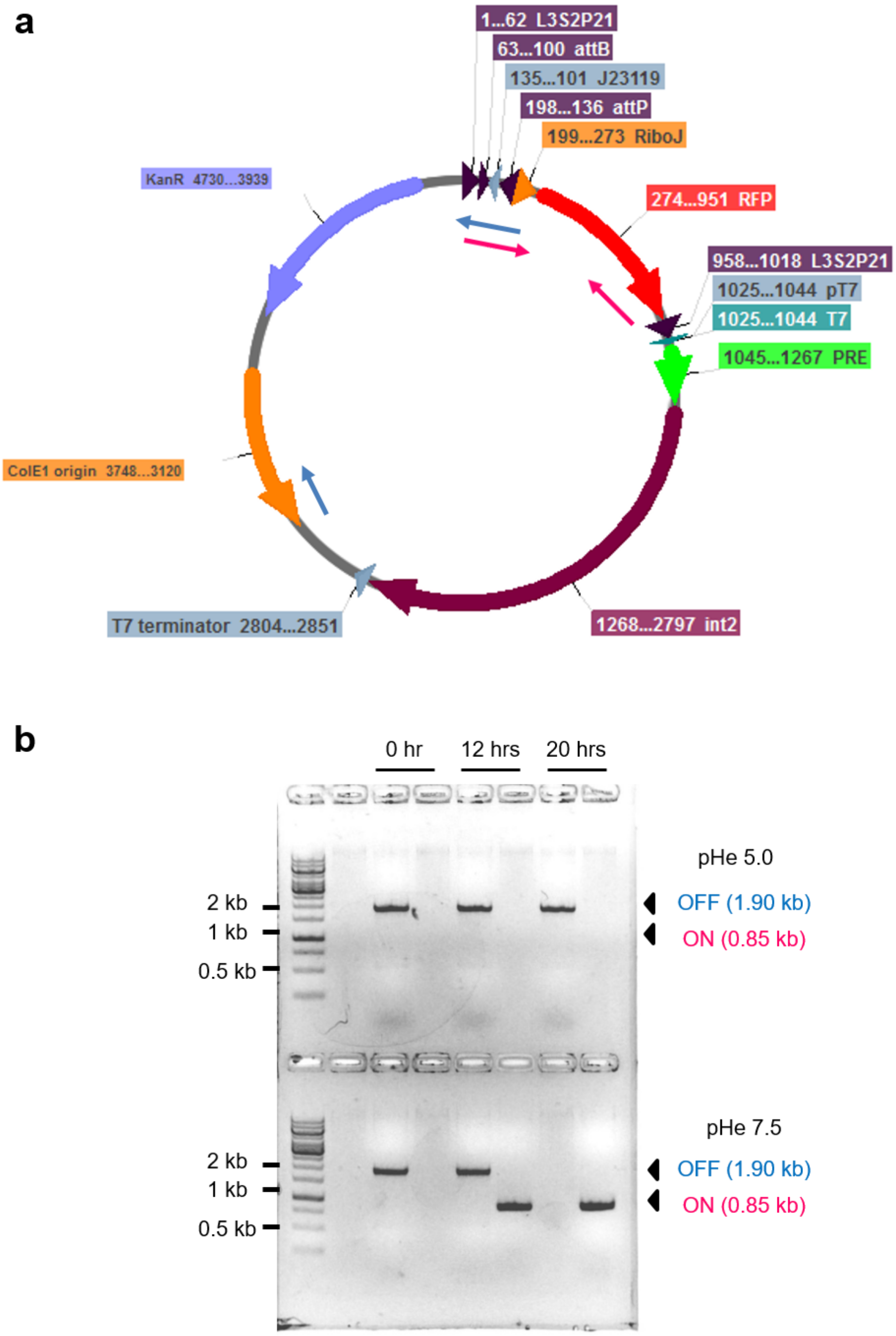

Supplementary Figure 10. PCR assay to characterize digital pH-sensing system. (**a**) Plasmid diagram of reporter plasmid of digital pH-sensing system. Blue and red arrows indicate the primers used to detect OFF (1.90 kb) and ON (0.85 kb) DNA configurations of the system. (**b**) Agarose gel showing the switch in DNA configurations between OFF and ON states at different pHe conditions. Top: pHe 5.0, bottom: pHe 7.5.

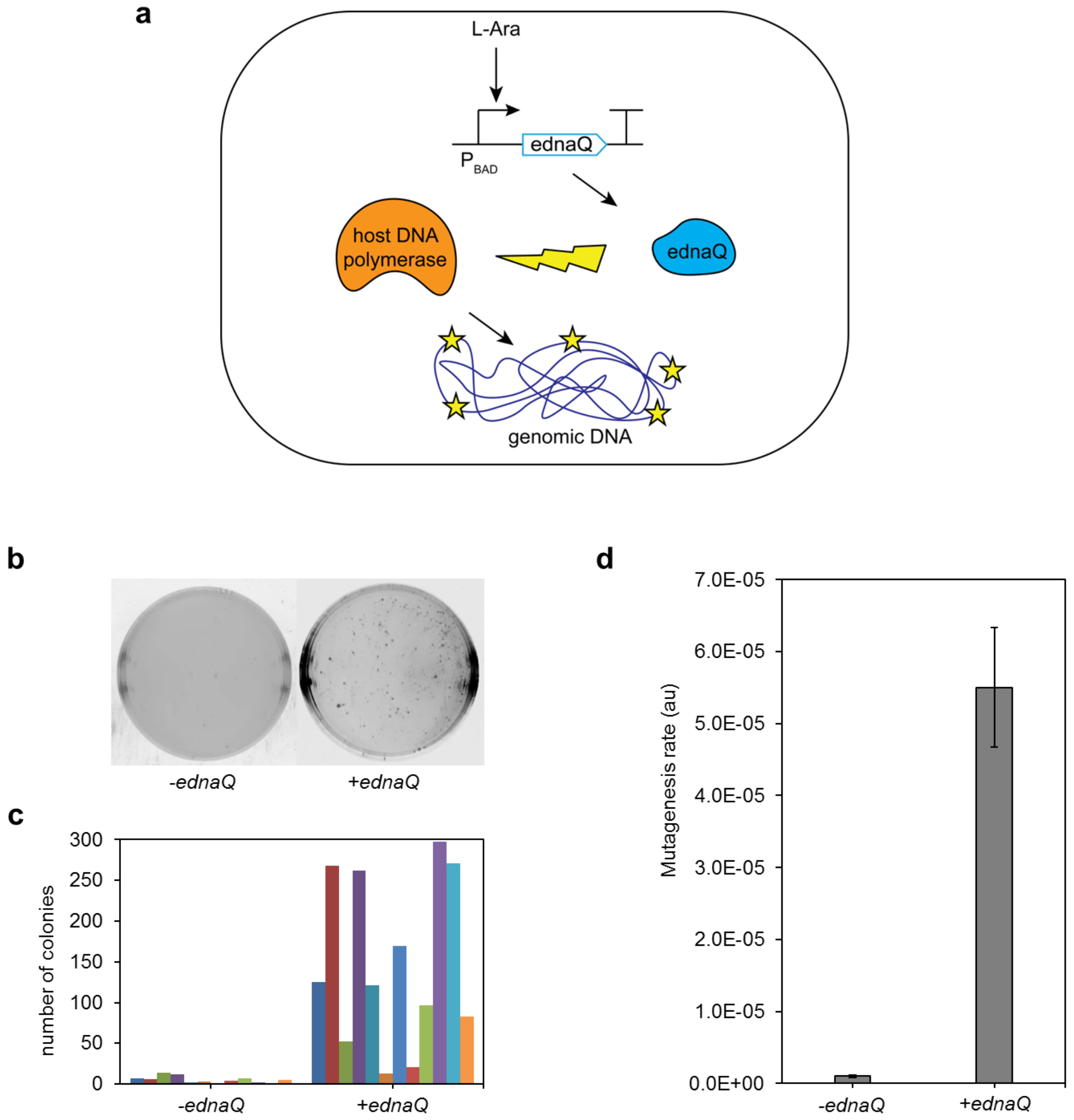

Supplementary Figure 11. Characterization of *ednaQ* genome mutator module. (**a**) Schematic of genetic construct used to characterize *ednaQ* function. (**b**) Visual inspection of rifampicin resistant colonies from reversion mutant assay. (**c**) Rifampicin resistant colonies obtained from different assay plates. (**d**) Genome mutation rates of cells without or with *ednaQ* induction. Data represents mean of twelve biological replicates.

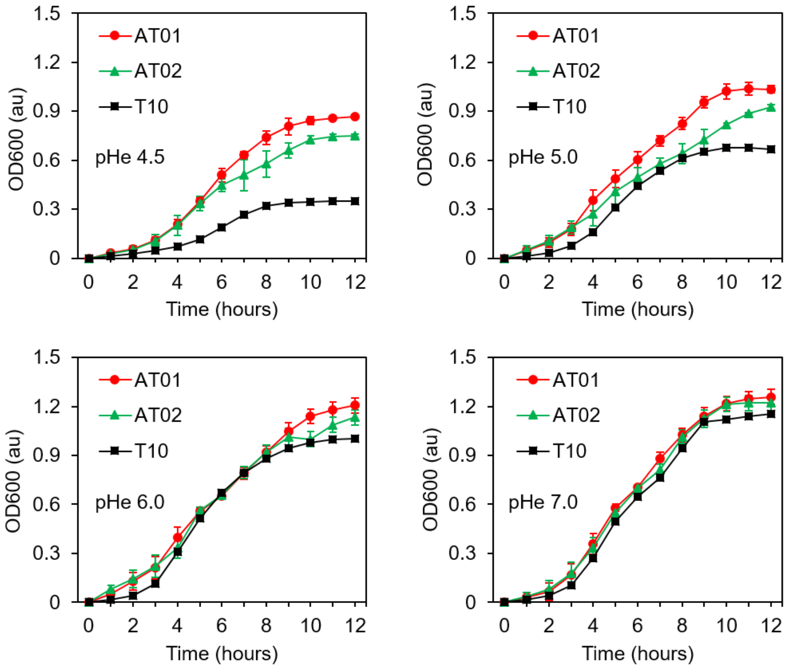

Supplementary Figure 12. Growth profiles of AT01, AT02, and T10 at varying pHe levels. Data represents mean of three biological replicates.

BioCyc ID	Gene name	Function
ECDH10B_0229	ykfH	hypothetical protein
ECDH10B_0232	ykfF	CP4-6 prophage; hypothetical protein
ECDH10B_2812	ypjJ	hypothetical protein

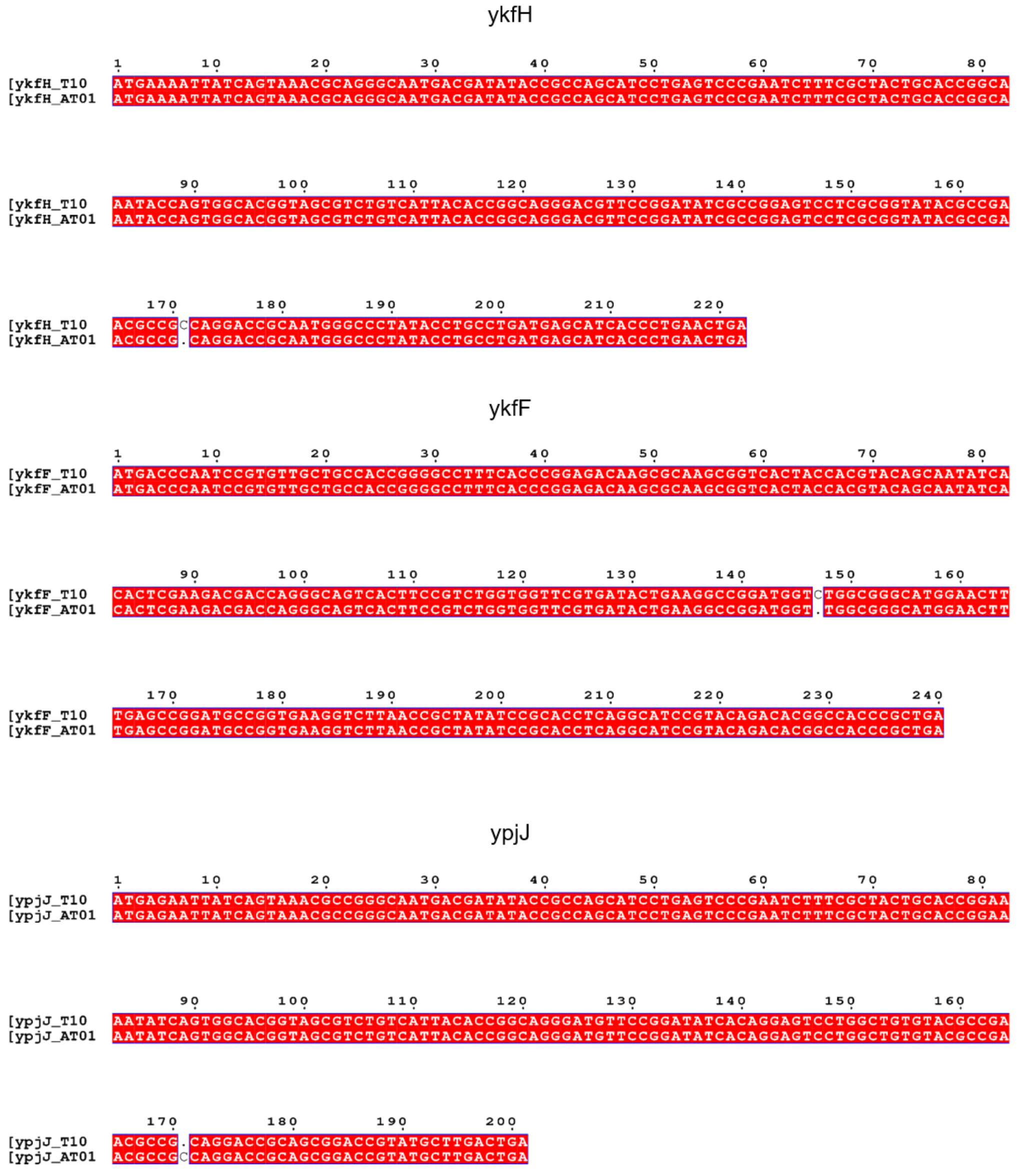

Supplementary Figure 13. Details of InDel modifications in AT01 genome comparing to T10. Sequence alignments of CDSs of ykfH, ykfF, and ypjJ in AT01 and T10 are shown.

a

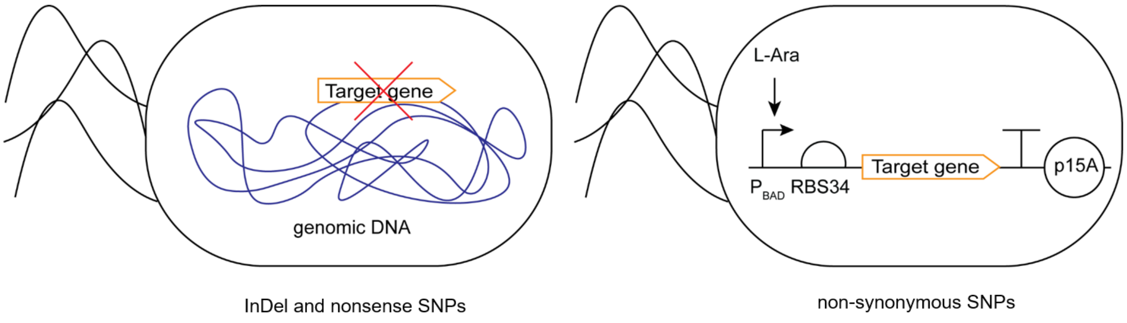

b

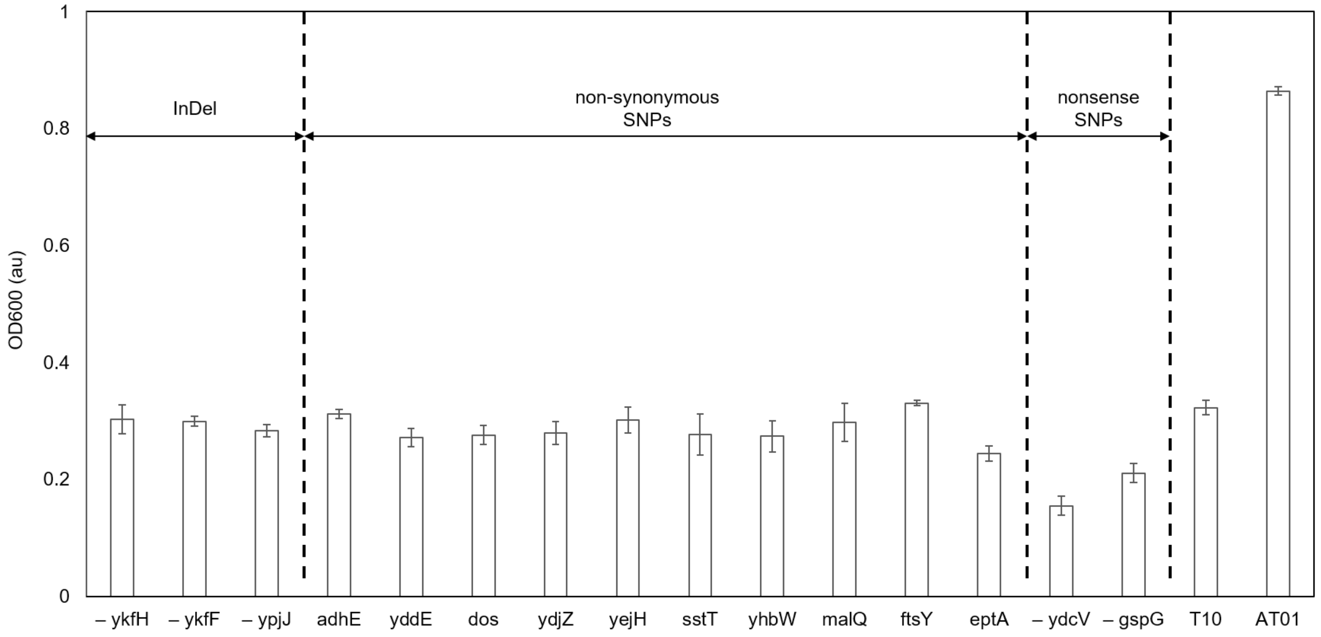

Supplementary Figure 14. Complementation assays to test for effects of modified genes in AT01 on acid-tolerant phenotype. (**a**) Constructs to test for effects of modified genes in AT01. For InDel (ykfH, ykfF, ypjJ) and nonsense SNPs (ydcV, gspG) modifications, target genes were deleted from parental strains. For non-synonymous SNPs (adhE, yddE, dos, ydjZ, yejH, sstT, yhbW, malQ, ftsY, eptA), target genes were overexpressed from medium copy plasmid (p15A origin) under control of PBAD promoter. (**b**) Stationary-phase cell density (OD600) of test strains, T10, and AT01 cultures growth at acidic condition (pHe 4.5) at 37°C for 12 hours are reported. (-) indicates gene deletion. Data represents mean of three biological replicates.

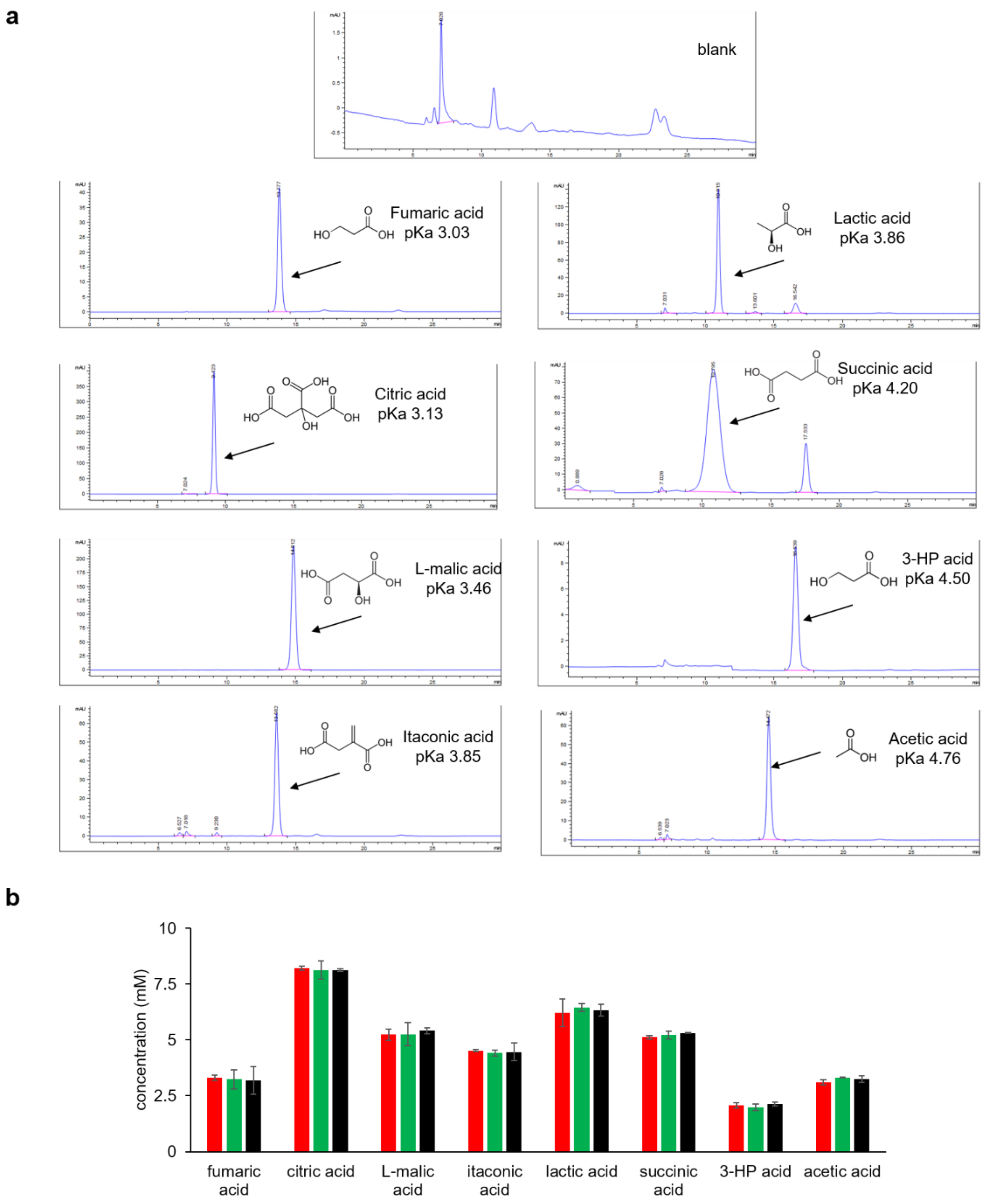

Supplementary Figure 15. HPLC analysis of intracellular supernatants of strains AT01, AT02, T10 subjected to organic acid challenge. In all experiments, 25mM of organic acids were added to the neutral M9 media containing initial cell inoculum (OD~0.01). After 12 hours of incubation, normalized cell cultures (~OD 0.3) were extracted to harvest intracellular environment. The intracellular supernatants were analyzed by HPLC and detected at 210 nm. (**a**) Chromatograms show the presence of organic acids in intracellular supernatants. (**b**) Quantification of the intracellular organic acid concentration of AT01 (red), AT02 (green), T10 (black). The concentration of intracellular organic acids in 1 mL samples extracted from normalized post-acid challenge cultures (OD600~1.0) are reported. Data represents mean of three biological replicates.

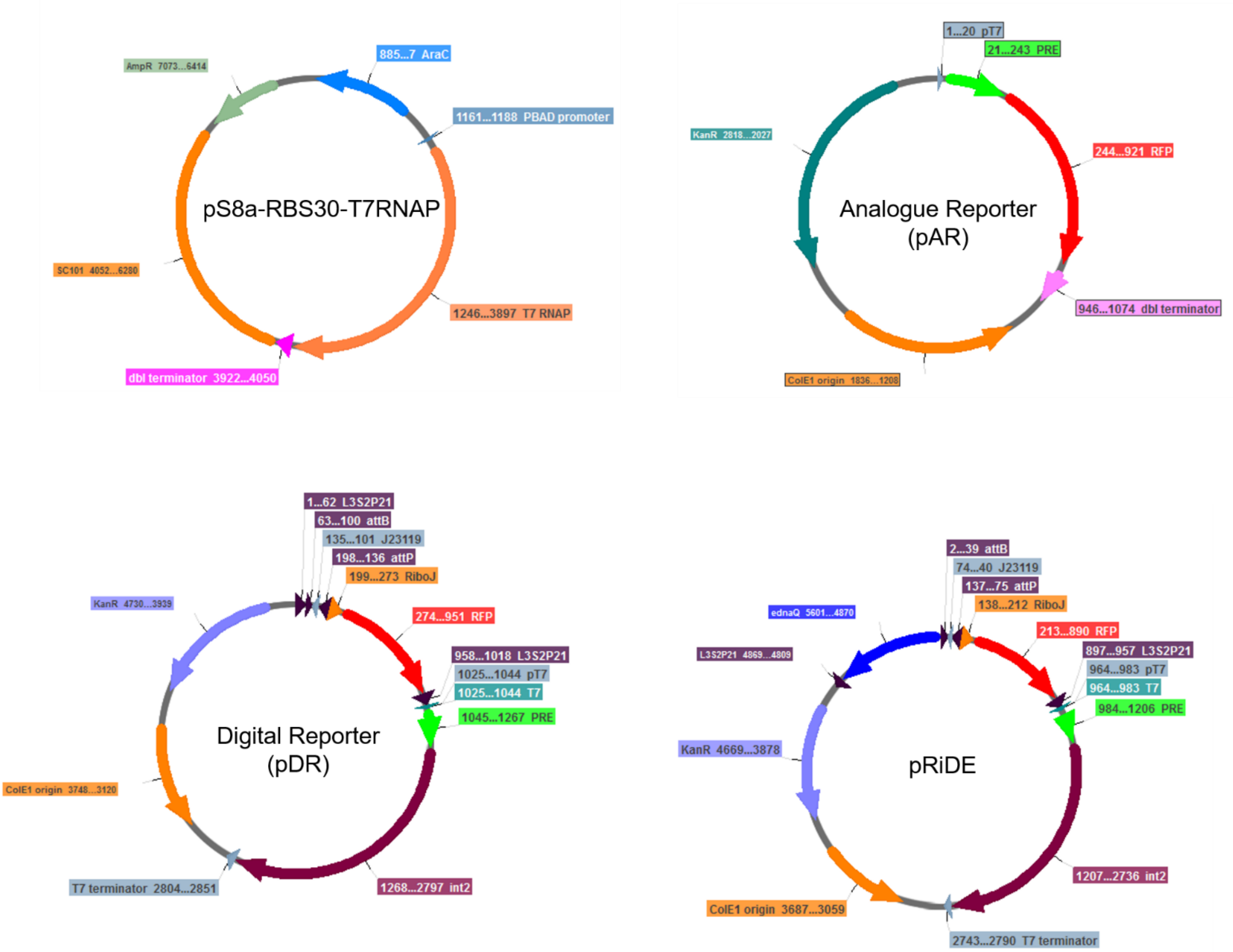

Supplementary Figure 16. Representative plasmid maps of genetic constructs used in this study.

Supplementary Tables

BioCyc ID	Mutation type	Gene name	Nucleotide mutation (T10<->AT01)	Translated amino acid position	Codon mutation (T10<->AT01)	Amino acid mutation (T10<->AT01)	Function
ECDH10B_1302	nonsyn	adhE	A<->G	255	GTT<->GCT	V<->A	fused acetaldehyde-CoA dehydrogenase and iron-dependent alcohol dehydrogenase and pyruvate-formate lyase deactivase
ECDH10B_1595	nonsyn	yddE	T<->C	139	AAC<->AGC	N<->S	conserved protein
ECDH10B_1620	nonsyn	dos	A<->G	425	TGG<->CGG	W<->R	cAMP phosphodiesterase%2C heme-regulated
ECDH10B_1890	nonsyn	ydjZ	A<->G	165	TAT<->TGT	Y<->C	conserved inner membrane protein
ECDH10B_2342	nonsyn	yejH	T<->C	584	TTA<->TCA	L<->S	predicted ATP-dependet helicase
ECDH10B_3265	nonsyn	sstT	G<->T	386	GCG<->TCG	A<->S	sodium:serine/threonine symporter
ECDH10B_3333	nonsyn	yhbW	A<->G	120	ACA<->GCA	T<->A	predicted enzyme
ECDH10B_3591	nonsyn	malQ	A<->G	344	TAT<->CAT	Y<->H	4-alpha-glucanotransferase (amylomaltase)
ECDH10B_3638	nonsyn	ftsY	G<->C	296	ATC<->ATG	I<->M	fused Signal Recognition Particle (SRP) receptor: membrane binding protein%3B conserved protein
ECDH10B_4306	nonsyn	eptA	T<->G	29	AAT<->CAT	N<->H	predicted metal dependent hydrolase
ECDH10B_1573	nonsense	ydcV	C<->T	92	CGA<->TGA	R<->X	predicted spermidine/putrescine transporter subunit%3B membrane component of ABC superfamily
ECDH10B_3503	nonsense	gspG	G<->T	128	GAA<->TAA	E<->X	pseudopilin%2C cryptic%2C general secretion pathway

Supplementary Table 1. Details of non-synonymous and nonsense SNPs modifications in AT01 genome comparing to T10.

Supplementary Table 2. List of plasmids used in this study.

Figure	Plasmids
1d	pS8a-RBS30-T7RNAP + pAR
2	pS8a-RBS30-T7RNAP + pAR
3a	pS8a-RBS30-T7RNAP + pDR
4a	pS8a-RBS30-T7RNAP + pRiDE
S2	pEk-J23119-RBS34-pHluorin2
S6	pS8a-RBS30-T7RNAP + pE7k-RFP
S9	pE8k-RBS34-ednaQ

Supplementary Table 3. Sequences of genetic parts used in this study. Red and lowercase letters indicate mutated nucleotides.

Parts	Sequence
PRE	TGCAAAGGGGAGTAACTTCATTGCCGGTCGATCGTCATTACGATGTGTGAAAAAACACATCCGGTCACCGGGCAACCCGAAAG<br>GAATACGCAGACGTATTCCTTTTTTGTTGTAAGTGAGACCTTGCCGGAAGGCGAGGTCTATGCATAAAAAGCAGCGGCTGACG<br>TCTTCCGACGTTGGCCGTTTTTTTATGTGTAAGGAACTTC
PREmR34	TGCAAAGGGGATTTCTCCTCTTTCTTCATTGCCGGTCGATCGTCATTACGATGTGTGAAAAAACACATCCGGTCACCGGGCAA<br>CCCGAAAGGAATACGCAGACGTATTCCTTTTTTGTTGTAAGTGAGACCTTGCCGGAAGGCGAGGTCTATGCATAAAAAGCAGC<br>GGCTGACGTCTTCCGACGTTGGCCGTTTTTTTATGTGTAAAAGAGGAGAAAACTTCT
PREmR34.1	TGCAAAGGGGATTTCTCCTCTAACTTCATTGCCGGTCGATCGTCATTACGATGTGTGAAAAAACACATCCGGTCACCGGGCAA<br>CCCGAAAGGAATACGCAGACGTATTCCTTTTTTGTTGTAAGTGAGACCTTGCCGGAAGGCGAGGTCTATGCATAAAAAGCAGC<br>GGCTGACGTCTTCCGACGTTGGCCGTTTTTTTATGTGTATTAGAGGAGAAAACTTCT
PREmR34.2	TGCAAAGGGGATAACTCCTCTTACTTCATTGCCGGTCGATCGTCATTACGATGTGTGAAAAAACACATCCGGTCACCGGGCAA<br>CCCGAAAGGAATACGCAGACGTATTCCTTTTTTGTTGTAAGTGAGACCTTGCCGGAAGGCGAGGTCTATGCATAAAAAGCAGC<br>GGCTGACGTCTTCCGACGTTGGCCGTTTTTTTATGTGTATAAGAGGAGTTAACTTCT
PREmR34.3	TGCAAAGGGGAAAACTCCTCAAACTTCATTGCCGGTCGATCGTCATTACGATGTGTGAAAAAACACATCCGGTCACCGGGCAA<br>CCCGAAAGGAATACGCAGACGTATTCCTTTTTTGTTGTAAGTGAGACCTTGCCGGAAGGCGAGGTCTATGCATAAAAAGCAGC<br>GGCTGACGTCTTCCGACGTTGGCCGTTTTTTTATGTGTATTTGAGGAGTTTACTTCT
T7WT	ATGAACACGATTAACATCGCTAAGAACGACTTCTCTGACATCGAACTGGCTGCTATCCCGTTCAACACTCTGGCTGACCATTA<br>CGGTGAGCGTTTAGCTCGCGAACAGTTGGCCCTTGAGCATGAGTCTTACGAGATGGGTGAAGCACGCTTCCGCAAGATGTTTG<br>AGCGTCAACTTAAAGCTGGTGAGGTTGCGGATAACGCTGCCGCCAAGCCTCTCATCACTACCCTACTCCCTAAGATGATTGCA<br>CGCATCAACGACTGGTTTGAGGAAGTGAAAGCTAAGCGCGGCAAGCGCCCGACAGCCTTCCAGTTCCTGCAAGAAATCAAGCC<br>GGAAGCCGTAGCGTACATCACCATTAAGACCACTCTGGCTTGCCTAACCAGTGCTGACAATACAACCGTTCAGGCTGTAGCAA<br>GCGCAATCGGTCGGGCCATTGAGGACGAGGCTCGCTTCGGTCGTATCCGTGACCTTGAAGCTAAGCACTTCAAGAAAAACGTT<br>GAGGAACAACTCAACAAGCGCGTAGGGCACGTCTACAAGAAAGCATTTATGCAAGTTGTCGAGGCTGACATGCTCTCTAAGGG<br>TCTACTCGGTGGCGAGGCGTGGTCTTCGTGGCATAAGGAAGACTCTATTCATGTAGGAGTACGCTGCATCGAGATGCTCATTG<br>AGTCAACCGGAATGGTTAGCTTACACCGCCAAAATGCTGGCGTAGTAGGTCAAGACTCTGAGACTATCGAACTCGCACCTGAA<br>TACGCTGAGGCTATCGCAACCCGTGCAGGTGCGCTGGCTGGCATCTCTCCGATGTTCCAACCTTGCGTAGTTCCTCCTAAGCC<br>GTGGACTGGCATTACTGGTGGTGGCTATTGGGCTAACGGTCGTCGTCCTCTGGCGCTGGTGCGTACTCACAGTAAGAAAGCAC<br>TGATGCGCTACGAAGACGTTTACATGCCTGAGGTGTACAAAGCGATTAACATTGCGCAAAACACCGCATGGAAAATCAACAAG<br>AAAGTCCTAGCGGTCGCCAACGTAATCACCAAGTGGAAGCATTGTCCGGTCGAGGACATCCCTGCGATTGAGCGTGAAGAACT<br>CCCGATGAAACCGGAAGACATCGACATGAATCCTGAGGCTCTCACCGCGTGGAAACGTGCTGCCGCTGCTGTGTACCGCAAGG<br>ACAAGGCTCGCAAGTCTCGCCGTATCAGCCTTGAGTTCATGCTTGAGCAAGCCAATAAGTTTGCTAACCATAAGGCCATCTGG<br>TTCCCTTACAACATGGACTGGCGCGGTCGTGTTTACGCTGTGTCAATGTTCAACCCGCAAGGTAACGATATGACCAAAGGACT<br>GCTTACGCTGGCGAAAGGTAAACCAATCGGTAAGGAAGGTTACTACTGGCTGAAAATCCACGGTGCAAACTGTGCGGGTGTCG<br>ATAAGGTTCCGTTCCCTGAGCGCATCAAGTTCATTGAGGAAAACCACGAGAACATCATGGCTTGCGCTAAGTCTCCACTGGAG<br>AACACTTGGTGGGCTGAGCAAGATTCTCCGTTCTGCTTCCTTGCGTTCTGCTTTGAGTACGCTGGGGTACAGCACCACGGCCT<br>GAGCTATAACTGCTCCCTTCCGCTGGCGTTTGACGGGTCTTGCTCTGGCATCCAGCACTTCTCCGCGATGCTCCGAGATGAGG<br>TAGGTGGTCGCGCGGTTAACTTGCTTCCTAGTGAAACCGTTCAGGACATCTACGGGATTGTTGCTAAGAAAGTCAACGAGATT<br>CTACAAGCAGACGCAATCAATGGGACCGATAACGAAGTAGTTACCGTGACCGATGAGAACACTGGTGAAATCTCTGAGAAAGT<br>CAAGCTGGGCACTAAGGCACTGGCTGGTCAATGGCTGGCTTACGGTGTTACTCGCAGTGTGACTAAGCGTTCAGTCATGACGC<br>TGGCTTACGGGTCCAAAGAGTTCGGCTTCCGTCAACAAGTGCTGGAAGATACCATTCAGCCAGCTATTGATTCCGGCAAGGGT<br>CTGATGTTCACTCAGCCGAATCAGGCTGCTGGATACATGGCTAAGCTGATTTGGGAATCTGTGAGCGTGACGGTGGTAGCTGC<br>GGTTGAAGCAATGAACTGGCTTAAGTCTGCTGCTAAGCTGCTGGCTGCTGAGGTCAAAGATAAGAAGACTGGAGAGATTCTTC<br>GCAAGCGTTGCGCTGTGCATTGGGTAACTCCTGATGGTTTCCCTGTGTGGCAGGAATACAAGAAGCCTATTCAGACGCGCTTG<br>AACCTGATGTTCCTCGGTCAGTTCCGCTTACAGCCTACCATTAACACCAACAAAGATAGCGAGATTGATGCACACAAACAGGA<br>GTCTGGTATCGCTCCTAACTTTGTACACAGCCAAGACGGTAGCCACCTTCGTAAGACTGTAGTGTGGGCACACGAGAAGTACG<br>GAATCGAATCTTTTGCACTGATTCACGACTCCTTCGGTACCATTCCGGCTGACGCTGCGAACCTGTTCAAAGCAGTGCGCGAA<br>ACTATGGTTGACACATATGAGTCTTGTGATGTACTGGCTGATTTCTACGACCAGTTCGCTGACCAGTTGCACGAGTCTCAATT<br>GGACAAAATGCCAGCACTTCCGGCTAAAGGTAACTTGAACCTCCGTGACATCTTAGAGTCGGACTTCGCGTTCGCGTAA

T7 Q49S	ATGAACACGATTAACATCGCTAAGAACGACTTCTCTGACATCGAACTGGCTGCTATCCCGTTCAACACTCTGGCTGACCATTA<br>CGGTGAGCGTTTAGCTCGCGAACAGTTGGCCCTTGAGCATGAGTCTTACGAGATGGGTGAAGCACGCTTCCGCAAGATGTTTG<br>AGCGTCAACTTAAAGCTGGTGAGGTTGCGGATAACGCTGCCGCCAAGCCTCTCATCACTACCCTACTCCCTAAGATGATTGCA<br>CGCATCAACGACTGGTTTGAGGAAGTGAAAGCTAAGCGCGGCAAGCGCCCGACAGCCTTCCAGTTCCTGCAAGAAATCAAGCC<br>GGAAGCCGTAGCGTACATCACCATTAAGACCACTCTGGCTTGCCTAACCAGTGCTGACAATACAACCGTTCAGGCTGTAGCAA<br>GCGCAATCGGTCGGGCCATTGAGGACGAGGCTCGCTTCGGTCGTATCCGTGACCTTGAAGCTAAGCACTTCAAGAAAAACGTT<br>GAGGAACAACTCAACAAGCGCGTAGGGCACGTCTACAAGAAAGCATTTATGCAAGTTGTCGAGGCTGACATGCTCTCTAAGGG<br>TCTACTCGGTGGCGAGGCGTGGTCTTCGTGGCATAAGGAAGACTCTATTCATGTAGGAGTACGCTGCATCGAGATGCTCATTG<br>AGTCAACCGGAATGGTTAGCTTACACCGCCAAAATGCTGGCGTAGTAGGTCAAGACTCTGAGACTATCGAACTCGCACCTGAA<br>TACGCTGAGGCTATCGCAACCCGTGCAGGTGCGCTGGCTGGCATCTCTCCGATGTTCCAACCTTGCGTAGTTCCTCCTAAGCC<br>GTGGACTGGCATTACTGGTGGTGGCTATTGGGCTAACGGTCGTCGTCCTCTGGCGCTGGTGCGTACTCACAGTAAGAAAGCAC<br>TGATGCGCTACGAAGACGTTTACATGCCTGAGGTGTACAAAGCGATTAACATTGCGCAAAACACCGCATGGAAAATCAACAAG<br>AAAGTCCTAGCGGTCGCCAACGTAATCACCAAGTGGAAGCATTGTCCGGTCGAGGACATCCCTGCGATTGAGCGTGAAGAACT<br>CCCGATGAAACCGGAAGACATCGACATGAATCCTGAGGCTCTCACCGCGTGGAAACGTGCTGCCGCTGCTGTGTACCGCAAGG<br>ACAAGGCTCGCAAGTCTCGCCGTATCAGCCTTGAGTTCATGCTTGAGCAAGCCAATAAGTTTGCTAACCATAAGGCCATCTGG<br>TTCCCTTACAACATGGACTGGCGCGGTCGTGTTTACGCTGTGTCAATGTTCAACCCGCAAGGTAACGATATGACCAAAGGACT<br>GCTTACGCTGGCGAAAGGTAAACCAATCGGTAAGGAAGGTTACTACTGGCTGAAAATCCACGGTGCAAACTGTGCGGGTGTCG<br>ATAAGGTTCCGTTCCCTGAGCGCATCAAGTTCATTGAGGAAAACCACGAGAACATCATGGCTTGCGCTAAGTCTCCACTGGAG<br>AACACTTGGTGGGCTGAGCAAGATTCTCCGTTCTGCTTCCTTGCGTTCTGCTTTGAGTACGCTGGGGTACAGCACCACGGCCT<br>GAGCTATAACTGCTCCCTTCCGCTGGCGTTTGACGGGTCTTGCTCTGGCATCCAGCACTTCTCCGCGATGCTCCGAGATGAGG<br>TAGGTGGTCGCGCGGTTAACTTGCTTCCTAGTGAAACCGTTCAGGACATCTACGGGATTGTTGCTAAGAAAGTCAACGAGATT<br>CTACAAGCAGACGCAATCAATGGGACCGATAACGAAGTAGTTACCGTGACCGATGAGAACACTGGTGAAATCTCTGAGAAAGT<br>CAAGCTGGGCACTAAGGCACTGGCTGGTCAATGGCTGGCTTACGGTGTTACTCGCAGTGTGACTAAGCGTTCAGTCATGACGC<br>TGGCTTACGGGTCCAAAGAGTTCGGCTTCCGTCAAagcGTGCTGGAAGATACCATTCAGCCAGCTATTGATTCCGGCAAGGGT<br>CTGATGTTCACTCAGCCGAATCAGGCTGCTGGATACATGGCTAAGCTGATTTGGGAATCTGTGAGCGTGACGGTGGTAGCTGC<br>GGTTGAAGCAATGAACTGGCTTAAGTCTGCTGCTAAGCTGCTGGCTGCTGAGGTCAAAGATAAGAAGACTGGAGAGATTCTTC<br>GCAAGCGTTGCGCTGTGCATTGGGTAACTCCTGATGGTTTCCCTGTGTGGCAGGAATACAAGAAGCCTATTCAGACGCGCTTG<br>AACCTGATGTTCCTCGGTCAGTTCCGCTTACAGCCTACCATTAACACCAACAAAGATAGCGAGATTGATGCACACAAACAGGA<br>GTCTGGTATCGCTCCTAACTTTGTACACAGCCAAGACGGTAGCCACCTTCGTAAGACTGTAGTGTGGGCACACGAGAAGTACG<br>GAATCGAATCTTTTGCACTGATTCACGACTCCTTCGGTACCATTCCGGCTGACGCTGCGAACCTGTTCAAAGCAGTGCGCGAA<br>ACTATGGTTGACACATATGAGTCTTGTGATGTACTGGCTGATTTCTACGACCAGTTCGCTGACCAGTTGCACGAGTCTCAATT<br>GGACAAAATGCCAGCACTTCCGGCTAAAGGTAACTTGAACCTCCGTGACATCTTAGAGTCGGACTTCGCGTTCGCGTAA

T7 F644A	ATGAACACGATTAACATCGCTAAGAACGACTTCTCTGACATCGAACTGGCTGCTATCCCGTTCAACACTCTGGCTGACCATTA<br>CGGTGAGCGTTTAGCTCGCGAACAGTTGGCCCTTGAGCATGAGTCTTACGAGATGGGTGAAGCACGCTTCCGCAAGATGTTTG<br>AGCGTCAACTTAAAGCTGGTGAGGTTGCGGATAACGCTGCCGCCAAGCCTCTCATCACTACCCTACTCCCTAAGATGATTGCA<br>CGCATCAACGACTGGTTTGAGGAAGTGAAAGCTAAGCGCGGCAAGCGCCCGACAGCCTTCCAGTTCCTGCAAGAAATCAAGCC<br>GGAAGCCGTAGCGTACATCACCATTAAGACCACTCTGGCTTGCCTAACCAGTGCTGACAATACAACCGTTCAGGCTGTAGCAA<br>GCGCAATCGGTCGGGCCATTGAGGACGAGGCTCGCTTCGGTCGTATCCGTGACCTTGAAGCTAAGCACTTCAAGAAAAACGTT<br>GAGGAACAACTCAACAAGCGCGTAGGGCACGTCTACAAGAAAGCATTTATGCAAGTTGTCGAGGCTGACATGCTCTCTAAGGG<br>TCTACTCGGTGGCGAGGCGTGGTCTTCGTGGCATAAGGAAGACTCTATTCATGTAGGAGTACGCTGCATCGAGATGCTCATTG<br>AGTCAACCGGAATGGTTAGCTTACACCGCCAAAATGCTGGCGTAGTAGGTCAAGACTCTGAGACTATCGAACTCGCACCTGAA<br>TACGCTGAGGCTATCGCAACCCGTGCAGGTGCGCTGGCTGGCATCTCTCCGATGTTCCAACCTTGCGTAGTTCCTCCTAAGCC<br>GTGGACTGGCATTACTGGTGGTGGCTATTGGGCTAACGGTCGTCGTCCTCTGGCGCTGGTGCGTACTCACAGTAAGAAAGCAC<br>TGATGCGCTACGAAGACGTTTACATGCCTGAGGTGTACAAAGCGATTAACATTGCGCAAAACACCGCATGGAAAATCAACAAG<br>AAAGTCCTAGCGGTCGCCAACGTAATCACCAAGTGGAAGCATTGTCCGGTCGAGGACATCCCTGCGATTGAGCGTGAAGAACT<br>CCCGATGAAACCGGAAGACATCGACATGAATCCTGAGGCTCTCACCGCGTGGAAACGTGCTGCCGCTGCTGTGTACCGCAAGG<br>ACAAGGCTCGCAAGTCTCGCCGTATCAGCCTTGAGTTCATGCTTGAGCAAGCCAATAAGTTTGCTAACCATAAGGCCATCTGG<br>TTCCCTTACAACATGGACTGGCGCGGTCGTGTTTACGCTGTGTCAATGTTCAACCCGCAAGGTAACGATATGACCAAAGGACT<br>GCTTACGCTGGCGAAAGGTAAACCAATCGGTAAGGAAGGTTACTACTGGCTGAAAATCCACGGTGCAAACTGTGCGGGTGTCG<br>ATAAGGTTCCGTTCCCTGAGCGCATCAAGTTCATTGAGGAAAACCACGAGAACATCATGGCTTGCGCTAAGTCTCCACTGGAG<br>AACACTTGGTGGGCTGAGCAAGATTCTCCGTTCTGCTTCCTTGCGTTCTGCTTTGAGTACGCTGGGGTACAGCACCACGGCCT<br>GAGCTATAACTGCTCCCTTCCGCTGGCGTTTGACGGGTCTTGCTCTGGCATCCAGCACTTCTCCGCGATGCTCCGAGATGAGG<br>TAGGTGGTCGCGCGGTTAACTTGCTTCCTAGTGAAACCGTTCAGGACATCTACGGGATTGTTGCTAAGAAAGTCAACGAGATT<br>CTACAAGCAGACGCAATCAATGGGACCGATAACGAAGTAGTTACCGTGACCGATGAGAACACTGGTGAAATCTCTGAGAAAGT<br>CAAGCTGGGCACTAAGGCACTGGCTGGTCAATGGCTGGCTTACGGTGTTACTCGCAGTGTGACTAAGCGTTCAGTCATGACGC<br>TGGCTTACGGGTCCAAAGAGgcCGGCTTCCGTCAACAAGTGCTGGAAGATACCATTCAGCCAGCTATTGATTCCGGCAAGGGT<br>CTGATGTTCACTCAGCCGAATCAGGCTGCTGGATACATGGCTAAGCTGATTTGGGAATCTGTGAGCGTGACGGTGGTAGCTGC<br>GGTTGAAGCAATGAACTGGCTTAAGTCTGCTGCTAAGCTGCTGGCTGCTGAGGTCAAAGATAAGAAGACTGGAGAGATTCTTC<br>GCAAGCGTTGCGCTGTGCATTGGGTAACTCCTGATGGTTTCCCTGTGTGGCAGGAATACAAGAAGCCTATTCAGACGCGCTTG<br>AACCTGATGTTCCTCGGTCAGTTCCGCTTACAGCCTACCATTAACACCAACAAAGATAGCGAGATTGATGCACACAAACAGGA<br>GTCTGGTATCGCTCCTAACTTTGTACACAGCCAAGACGGTAGCCACCTTCGTAAGACTGTAGTGTGGGCACACGAGAAGTACG<br>GAATCGAATCTTTTGCACTGATTCACGACTCCTTCGGTACCATTCCGGCTGACGCTGCGAACCTGTTCAAAGCAGTGCGCGAA<br>ACTATGGTTGACACATATGAGTCTTGTGATGTACTGGCTGATTTCTACGACCAGTTCGCTGACCAGTTGCACGAGTCTCAATT<br>GGACAAAATGCCAGCACTTCCGGCTAAAGGTAACTTGAACCTCCGTGACATCTTAGAGTCGGACTTCGCGTTCGCGTAA

T7 G645A	ATGAACACGATTAACATCGCTAAGAACGACTTCTCTGACATCGAACTGGCTGCTATCCCGTTCAACACTCTGGCTGACCATTA<br>CGGTGAGCGTTTAGCTCGCGAACAGTTGGCCCTTGAGCATGAGTCTTACGAGATGGGTGAAGCACGCTTCCGCAAGATGTTTG<br>AGCGTCAACTTAAAGCTGGTGAGGTTGCGGATAACGCTGCCGCCAAGCCTCTCATCACTACCCTACTCCCTAAGATGATTGCA<br>CGCATCAACGACTGGTTTGAGGAAGTGAAAGCTAAGCGCGGCAAGCGCCCGACAGCCTTCCAGTTCCTGCAAGAAATCAAGCC<br>GGAAGCCGTAGCGTACATCACCATTAAGACCACTCTGGCTTGCCTAACCAGTGCTGACAATACAACCGTTCAGGCTGTAGCAA<br>GCGCAATCGGTCGGGCCATTGAGGACGAGGCTCGCTTCGGTCGTATCCGTGACCTTGAAGCTAAGCACTTCAAGAAAAACGTT<br>GAGGAACAACTCAACAAGCGCGTAGGGCACGTCTACAAGAAAGCATTTATGCAAGTTGTCGAGGCTGACATGCTCTCTAAGGG<br>TCTACTCGGTGGCGAGGCGTGGTCTTCGTGGCATAAGGAAGACTCTATTCATGTAGGAGTACGCTGCATCGAGATGCTCATTG<br>AGTCAACCGGAATGGTTAGCTTACACCGCCAAAATGCTGGCGTAGTAGGTCAAGACTCTGAGACTATCGAACTCGCACCTGAA<br>TACGCTGAGGCTATCGCAACCCGTGCAGGTGCGCTGGCTGGCATCTCTCCGATGTTCCAACCTTGCGTAGTTCCTCCTAAGCC<br>GTGGACTGGCATTACTGGTGGTGGCTATTGGGCTAACGGTCGTCGTCCTCTGGCGCTGGTGCGTACTCACAGTAAGAAAGCAC<br>TGATGCGCTACGAAGACGTTTACATGCCTGAGGTGTACAAAGCGATTAACATTGCGCAAAACACCGCATGGAAAATCAACAAG<br>AAAGTCCTAGCGGTCGCCAACGTAATCACCAAGTGGAAGCATTGTCCGGTCGAGGACATCCCTGCGATTGAGCGTGAAGAACT<br>CCCGATGAAACCGGAAGACATCGACATGAATCCTGAGGCTCTCACCGCGTGGAAACGTGCTGCCGCTGCTGTGTACCGCAAGG<br>ACAAGGCTCGCAAGTCTCGCCGTATCAGCCTTGAGTTCATGCTTGAGCAAGCCAATAAGTTTGCTAACCATAAGGCCATCTGG<br>TTCCCTTACAACATGGACTGGCGCGGTCGTGTTTACGCTGTGTCAATGTTCAACCCGCAAGGTAACGATATGACCAAAGGACT<br>GCTTACGCTGGCGAAAGGTAAACCAATCGGTAAGGAAGGTTACTACTGGCTGAAAATCCACGGTGCAAACTGTGCGGGTGTCG<br>ATAAGGTTCCGTTCCCTGAGCGCATCAAGTTCATTGAGGAAAACCACGAGAACATCATGGCTTGCGCTAAGTCTCCACTGGAG<br>AACACTTGGTGGGCTGAGCAAGATTCTCCGTTCTGCTTCCTTGCGTTCTGCTTTGAGTACGCTGGGGTACAGCACCACGGCCT<br>GAGCTATAACTGCTCCCTTCCGCTGGCGTTTGACGGGTCTTGCTCTGGCATCCAGCACTTCTCCGCGATGCTCCGAGATGAGG<br>TAGGTGGTCGCGCGGTTAACTTGCTTCCTAGTGAAACCGTTCAGGACATCTACGGGATTGTTGCTAAGAAAGTCAACGAGATT<br>CTACAAGCAGACGCAATCAATGGGACCGATAACGAAGTAGTTACCGTGACCGATGAGAACACTGGTGAAATCTCTGAGAAAGT<br>CAAGCTGGGCACTAAGGCACTGGCTGGTCAATGGCTGGCTTACGGTGTTACTCGCAGTGTGACTAAGCGTTCAGTCATGACGC<br>TGGCTTACGGGTCCAAAGAGTTCGcCTTCCGTCAACAAGTGCTGGAAGATACCATTCAGCCAGCTATTGATTCCGGCAAGGGT<br>CTGATGTTCACTCAGCCGAATCAGGCTGCTGGATACATGGCTAAGCTGATTTGGGAATCTGTGAGCGTGACGGTGGTAGCTGC<br>GGTTGAAGCAATGAACTGGCTTAAGTCTGCTGCTAAGCTGCTGGCTGCTGAGGTCAAAGATAAGAAGACTGGAGAGATTCTTC<br>GCAAGCGTTGCGCTGTGCATTGGGTAACTCCTGATGGTTTCCCTGTGTGGCAGGAATACAAGAAGCCTATTCAGACGCGCTTG<br>AACCTGATGTTCCTCGGTCAGTTCCGCTTACAGCCTACCATTAACACCAACAAAGATAGCGAGATTGATGCACACAAACAGGA<br>GTCTGGTATCGCTCCTAACTTTGTACACAGCCAAGACGGTAGCCACCTTCGTAAGACTGTAGTGTGGGCACACGAGAAGTACG<br>GAATCGAATCTTTTGCACTGATTCACGACTCCTTCGGTACCATTCCGGCTGACGCTGCGAACCTGTTCAAAGCAGTGCGCGAA<br>ACTATGGTTGACACATATGAGTCTTGTGATGTACTGGCTGATTTCTACGACCAGTTCGCTGACCAGTTGCACGAGTCTCAATT<br>GGACAAAATGCCAGCACTTCCGGCTAAAGGTAACTTGAACCTCCGTGACATCTTAGAGTCGGACTTCGCGTTCGCGTAA

T7 FQ	ATGAACACGATTAACATCGCTAAGAACGACTTCTCTGACATCGAACTGGCTGCTATCCCGTTCAACACTCTGGCTGACCATTA<br>CGGTGAGCGTTTAGCTCGCGAACAGTTGGCCCTTGAGCATGAGTCTTACGAGATGGGTGAAGCACGCTTCCGCAAGATGTTTG<br>AGCGTCAACTTAAAGCTGGTGAGGTTGCGGATAACGCTGCCGCCAAGCCTCTCATCACTACCCTACTCCCTAAGATGATTGCA<br>CGCATCAACGACTGGTTTGAGGAAGTGAAAGCTAAGCGCGGCAAGCGCCCGACAGCCTTCCAGTTCCTGCAAGAAATCAAGCC<br>GGAAGCCGTAGCGTACATCACCATTAAGACCACTCTGGCTTGCCTAACCAGTGCTGACAATACAACCGTTCAGGCTGTAGCAA<br>GCGCAATCGGTCGGGCCATTGAGGACGAGGCTCGCTTCGGTCGTATCCGTGACCTTGAAGCTAAGCACTTCAAGAAAAACGTT<br>GAGGAACAACTCAACAAGCGCGTAGGGCACGTCTACAAGAAAGCATTTATGCAAGTTGTCGAGGCTGACATGCTCTCTAAGGG<br>TCTACTCGGTGGCGAGGCGTGGTCTTCGTGGCATAAGGAAGACTCTATTCATGTAGGAGTACGCTGCATCGAGATGCTCATTG<br>AGTCAACCGGAATGGTTAGCTTACACCGCCAAAATGCTGGCGTAGTAGGTCAAGACTCTGAGACTATCGAACTCGCACCTGAA<br>TACGCTGAGGCTATCGCAACCCGTGCAGGTGCGCTGGCTGGCATCTCTCCGATGTTCCAACCTTGCGTAGTTCCTCCTAAGCC<br>GTGGACTGGCATTACTGGTGGTGGCTATTGGGCTAACGGTCGTCGTCCTCTGGCGCTGGTGCGTACTCACAGTAAGAAAGCAC<br>TGATGCGCTACGAAGACGTTTACATGCCTGAGGTGTACAAAGCGATTAACATTGCGCAAAACACCGCATGGAAAATCAACAAG<br>AAAGTCCTAGCGGTCGCCAACGTAATCACCAAGTGGAAGCATTGTCCGGTCGAGGACATCCCTGCGATTGAGCGTGAAGAACT<br>CCCGATGAAACCGGAAGACATCGACATGAATCCTGAGGCTCTCACCGCGTGGAAACGTGCTGCCGCTGCTGTGTACCGCAAGG<br>ACAAGGCTCGCAAGTCTCGCCGTATCAGCCTTGAGTTCATGCTTGAGCAAGCCAATAAGTTTGCTAACCATAAGGCCATCTGG<br>TTCCCTTACAACATGGACTGGCGCGGTCGTGTTTACGCTGTGTCAATGTTCAACCCGCAAGGTAACGATATGACCAAAGGACT<br>GCTTACGCTGGCGAAAGGTAAACCAATCGGTAAGGAAGGTTACTACTGGCTGAAAATCCACGGTGCAAACTGTGCGGGTGTCG<br>ATAAGGTTCCGTTCCCTGAGCGCATCAAGTTCATTGAGGAAAACCACGAGAACATCATGGCTTGCGCTAAGTCTCCACTGGAG<br>AACACTTGGTGGGCTGAGCAAGATTCTCCGTTCTGCTTCCTTGCGTTCTGCTTTGAGTACGCTGGGGTACAGCACCACGGCCT<br>GAGCTATAACTGCTCCCTTCCGCTGGCGTTTGACGGGTCTTGCTCTGGCATCCAGCACTTCTCCGCGATGCTCCGAGATGAGG<br>TAGGTGGTCGCGCGGTTAACTTGCTTCCTAGTGAAACCGTTCAGGACATCTACGGGATTGTTGCTAAGAAAGTCAACGAGATT<br>CTACAAGCAGACGCAATCAATGGGACCGATAACGAAGTAGTTACCGTGACCGATGAGAACACTGGTGAAATCTCTGAGAAAGT<br>CAAGCTGGGCACTAAGGCACTGGCTGGTCAATGGCTGGCTTACGGTGTTACTCGCAGTGTGACTAAGCGTTCAGTCATGACGC<br>TGGCTTACGGGTCCAAAGAGgcCGGCTTCCGTCAAagcGTGCTGGAAGATACCATTCAGCCAGCTATTGATTCCGGCAAGGGT<br>CTGATGTTCACTCAGCCGAATCAGGCTGCTGGATACATGGCTAAGCTGATTTGGGAATCTGTGAGCGTGACGGTGGTAGCTGC<br>GGTTGAAGCAATGAACTGGCTTAAGTCTGCTGCTAAGCTGCTGGCTGCTGAGGTCAAAGATAAGAAGACTGGAGAGATTCTTC<br>GCAAGCGTTGCGCTGTGCATTGGGTAACTCCTGATGGTTTCCCTGTGTGGCAGGAATACAAGAAGCCTATTCAGACGCGCTTG<br>AACCTGATGTTCCTCGGTCAGTTCCGCTTACAGCCTACCATTAACACCAACAAAGATAGCGAGATTGATGCACACAAACAGGA<br>GTCTGGTATCGCTCCTAACTTTGTACACAGCCAAGACGGTAGCCACCTTCGTAAGACTGTAGTGTGGGCACACGAGAAGTACG<br>GAATCGAATCTTTTGCACTGATTCACGACTCCTTCGGTACCATTCCGGCTGACGCTGCGAACCTGTTCAAAGCAGTGCGCGAA<br>ACTATGGTTGACACATATGAGTCTTGTGATGTACTGGCTGATTTCTACGACCAGTTCGCTGACCAGTTGCACGAGTCTCAATT<br>GGACAAAATGCCAGCACTTCCGGCTAAAGGTAACTTGAACCTCCGTGACATCTTAGAGTCGGACTTCGCGTTCGCGTAA

pT7	TAATACGACTCACTATAGGGAGA
pT7m1	TAATACGACTCACTAcaggcAGA
pT7m2	TAATACGACTCACTAgagagAGA
pT7m3	TAATACGACTCACTAatgggAGA
pT7m4	TAATACGACTCACTAtaggtAGA
int2	ATGCCGATTGCACCGGAATTTCTGAGCCTGGCATATCCGGGTCAAGAATTTCCGGCATATCTGTATGGTCGTGCAAGCCGTGA<br>TCCGAAACGTAAAGGTCGTAGCGTTCAGAGCCAGCTGGATGAAGGTCGTGCCACCTGTCTGGATGCAGGTTGGCCGATTGCCG<br>GTGAATTTAAAGATGTTGATCGTAGCGCAAGCGCATATGCACGTCGTACCCGTGATGAATTTGAAGAAATGATTGCAGGTATT<br>CAGGCAGGCGAATGTCGTATTCTGGTTGCATTTGAAGCAAGCCGTTATTATCGTGATCTGGAAGCATATGTTCGTCTGCGTCG<br>TGTTTGTCGTGAAGCCGGTGTTCTGCTGTGTTATAATGGTCAGGTTTATGATCTGAGCAAAAGCGCAGATCGTAAAGCAACCG<br>CACAGGATGCAGTTAATGCCGAAGGTGAAGCAGATGATATTCGTGAACGTAATCTGCGTACCACCCGTCTGAATGCAAAACGT<br>GGTGGTGCACATGGTCCGGTTCCGGATGGTTATAAACGTCGTTATGATCCGGATAGCGGTGATCTGGTTGATCAGATTCCGCA<br>TCCTGATCGTGCAGGTCTGATTACCGAAATTTTTCGTCGTGCAGCAGCAGCCGAACCGCTGGCAGCAATTTGCCGTGATCTGA<br>ATGAACGTGGTGAAACCACCCATCGTGGTAAAGCATGGCAGCGTCATCATCTGCATGCAATTCTGCGTAATCCTGCATATATT<br>GGTCATCGTCGTCATCTGGGTGTTGATACCGGTAAAGGTATGTGGGCACCGATTTGTGATGATGAAGATTTTGCAGAAACCTT<br>TCAGGCCGTTCAAGAAATTCTGAGTCTGCCTGGTCGTCAGCTGAGTCCGGGTCCGGAAGCACAGCATCTGCAGACCGGTATTG<br>CACTGTGTGGTGAACATCCTGATGAACCGCCTCTGCGTAGCGTTACCGTTCGTGGTCGTACCAATTATAACTGTAGCACCCGT<br>TATGATGTTGCCATGCGTGAAGATCGTATGGATGCATTTGTTGAAGAAAGCGTGATTACCTGGCTGGCAAGTGATGAAGCAGT<br>TGCAGCCTTTGAAGATAACACCGATGATGAACGTACCCGTAAAGCACGTATTCGTCTGAAAGTTCTGGAAGAACAGCTGGAAG<br>CAGCACAGAAACAGGCACGTACCCTGCGTCCGGATGGCATGGGTATGCTGCTGAGCATTGATAGCCTGGCAGGTCTGGAAGCC<br>GAACTGACACCGCAGATTGATAAAGCCCGTCAAGAAAGCCGTAGCCTGCATGTTCCGGCACTGCTGCGTGATCTGCTGGGTAA<br>ACCGCGTGCAGATGTGGATCGTGCATGGAATGAAGCACTGACCCTGCCGCAGCGTCGCATGATTCTGCGCATGGTTGTTACCA<br>TTCGTCTGTTTAAAGCAGGTAGCCGTGGTGTTCGTGCAATTGAACCGGGTCGTATTACCCTGAGCTATGTTGGCGAACCGGGT<br>TTTAAACCGGTTGGTGGTAATCGTGCAAAACAGTAA
int2B	GGACGGCGCAGAAGGGGAGTAGCTCTTCGCCGGACCGTCGACATACTGCTCAGCTCGTC
int2P	GCTCATGTATGTGTCTACGCGAGATTCTCGCCCGAGAACTTCTGCAAGGCACTGCTCTTGGCT
RiboJ	AGCTGTCACCGGATGTGCTTTCCGGTCTGATGAGTCCGTGAGGACGAAACAGCCTCTACAAATAATTTTGTTTAA
RFP	ATGGCGAGTAGCGAAGACGTTATCAAAGAGTTCATGCGTTTCAAAGTTCGTATGGAAGGTTCCGTTAACGGTCACGAGTTCGA<br>AATCGAAGGTGAAGGTGAAGGTCGTCCGTACGAAGGTACCCAGACCGCTAAACTGAAAGTTACCAAAGGTGGTCCGCTGCCGT<br>TCGCTTGGGACATCCTGTCCCCGCAGTTCCAGTACGGTTCCAAAGCTTACGTTAAACACCCGGCTGACATCCCGGACTACCTG<br>AAACTGTCCTTCCCGGAAGGTTTCAAATGGGAACGTGTTATGAACTTCGAAGACGGTGGTGTTGTTACCGTTACCCAGGACTC<br>CTCCCTGCAAGACGGTGAGTTCATCTACAAAGTTAAACTGCGTGGTACCAACTTCCCGTCCGACGGTCCGGTTATGCAGAAAA<br>AAACCATGGGTTGGGAAGCTTCCACCGAACGTATGTACCCGGAAGACGGTGCTCTGAAAGGTGAAATCAAAATGCGTCTGAAA<br>CTGAAAGACGGTGGTCACTACGACGCTGAAGTTAAAACCACCTACATGGCTAAAAAACCGGTTCAGCTGCCGGGTGCTTACAA<br>AACCGACATCAAACTGGACATCACCTCCCACAACGAAGACTACACCATCGTTGAACAGTACGAACGTGCTGAAGGTCGTCACT<br>CCACCGGTGCTTAA
ednaQ	ATGAGCACTGCAATTACACGCCAGATCGTTCTCGATACCGAAAtCACCGGTATGAACCAGATTGGTGCGCACTATGAAGGCCA<br>CAAGATCATTGAGATTGGTGCCGTTGAAGTGGTGAACCGTCGCCTGACGGGCAATAACTTCCATGTTTATCTCAAACCCGATC<br>GGCTGGTGGAcCCGGAAGCCTTTGGCGTACATGGTATTGCCGATGAATTTTgGCTCGATAAGCCCACGTTTGCCGAAGTAGCC<br>GATGAGTTCATGGACTATATTCGCGGCGCGGAGTTGGTGATCCATAACGCAGCGTTCGATATCGGCTTTATGGACTACGAGTT<br>TTCGTTGCTTAAGCGCGATATTCCGAAGACCAATACTTTCTGTAAGGTCACCGATAGCCTTGCGGTGGCGAGGAAAATGTTTC<br>CCGGTAAGCGCAACAGCCTCGATGCGTTATGTGCTCGCTACGAAATAGATAACAGTAAACGAACGCgtCACGGGGtgTTACTC<br>GATGCCCAGATCCTTGCGGAAGTTTATCTGGCGATGACCGGTGGTCAAACGTCGATGGCTTTTGCGATGGAAGGAGAGACACA<br>ACAGCAACAAGGTGAAGCAACAATTCAGCGCATTGTACGTCAGGCAAGTAAGTTACGCGTTGTTTTTGCGACAGATGAtGAGc<br>tgGCAGCTCATGAAGCCCGTCTCGATCTGGTGCAGAAGAAAGGCGGAAGTTGCCTCTGGCGAGCATAA
J23119	TTGATTGACAGCTAGCTCAGTCCTAGGTATAATGCTAGC
pHluorin2	ATGAGTAAAGGGGAGGAACTGTTTACTGGTGTTGTTCCGATTCTGGTGGAACTGGACGGAGACGTCAACGGGCATAAATTCTC<br>GGTCTCAGGAGAAGGTGAGGGCGATGCCACATATGGAAAATTAACCTTAAAATTCATCTGTACCACGGGCAAACTGCCGGTAC<br>CATGGCCGACCCTGGTGACGACACTCAGTTATGGTGTCCAGTGCTTCTCTCGCTATCCAGATCACATGAAGCAGCACGACTTT<br>TTTAAGTCAGCGATGCCTGAAGGTTACGTCCAGGAACGTACCATCTTTTTTAAAGATGATGGCAATTATAAAACTCGTGCGGA<br>AGTAAAGTTTGAAGGTGATACCCTGGTAAACCGCATTGAACTCAAGGGCATTGACTTCAAAGAAGATGGTAACATTCTCGGCC<br>ACAAGCTGGAGTACAATTACAACGAACATTTAGTGTATATTATGGCCGATAAGCAGAAAAATGGAACAAAGGCGATTTTCCAG<br>GTGCATCATAACATCGAGGATGGCAGCGTGCAACTGGCAGATCATTACCAACAGAACACACCAATTGGCGACGGTCCGGTTTT<br>ATTGCCGGATAACCATTATCTGCATACGCAATCCGCGTTGTCGAAAGATCCGAATGAGAAGCGCGATCACATGGTACTGCTGG<br>AGTTTGTTACTGCAGCAGGGATCACACATGGAATGGACGAGCTGTACAAGTAA
